# Expanding the Scope of Immunotherapy in Colorectal Cancer: Current Clinical Approaches and Future Directions

**DOI:** 10.1155/2020/9037217

**Published:** 2020-01-25

**Authors:** Malek Kreidieh, Deborah Mukherji, Sally Temraz, Ali Shamseddine

**Affiliations:** Division of Hematology and Oncology, Department of Internal Medicine, American University of Beirut, Beirut, Lebanon

## Abstract

The success of immune checkpoint inhibitors (ICIs) in an increasing range of heavily mutated tumor types such as melanoma has culminated in their exploration in different subsets of patients with metastatic colorectal cancer (mCRC). As a result of their dramatic and durable response rates in patients with chemorefractory, mismatch repair-deficient-microsatellite instability-high (dMMR-MSI-H) mCRC, ICIs have become potential alternatives to classical systemic therapies. The anti-programmed death-1 (PD-1) agents, Pembrolizumab and Nivolumab, have been granted FDA approval for this subset of patients. Unfortunately, however, not all CRC cases with the dMMR-MSI-H phenotype respond well to ICIs, and ongoing studies are currently exploring biomarkers that can predict good response to them. Another challenge lies in developing novel treatment strategies for the subset of patients with the mismatch repair-proficient-microsatellite instability-low (pMMR-MSI-L) phenotype that comprises 95% of all mCRC cases in whom treatment with currently approved ICIs has been largely unsuccessful. Approaches aiming at overcoming the resistance of tumors in this subset of patients are being developed including combining different checkpoint inhibitors with either chemotherapy, anti-angiogenic agents, cancer vaccines, adoptive cell transfer (ACT), or bispecific T-cell (BTC) antibodies. This review describes the rationale behind using immunotherapeutics in CRC. It sheds light on the progress made in the use of immunotherapy in the treatment of patients with dMMR-MSI-H CRC. It also discusses emerging approaches and proposes potential strategies for targeting the immune microenvironment in patients with pMMR-MSI-L CRC tumors in an attempt to complement immune checkpoint inhibition.

## 1. Introduction

### 1.1. Colorectal Cancer-Epidemiology and Prognosis

Colorectal cancer (CRC) is the third most common malignancy worldwide with about 1.4 million newly diagnosed cases per year [1, 2]. It is the third leading cause of cancer-related mortality in the United States and is responsible for around 700,000 annual deaths worldwide [2]. By the year 2030, the projected global burden of CRC is expected to increase by 60% [1].

Although preventive and screening strategies have been appropriately developed in several countries, around 25% of patients still present at late stages, and 25–50% of them present at an early stage but proceed to develop regional or distant metastasis later on [2, 3–8]. Among those presenting with late stage disease, 86% die within 5 years [9]. Despite advances in systemic therapy and liver-directed treatments, the prognosis of patients with metastatic CRC (mCRC) remains poor, with a low median survival ranging between 5 months and 2 years and a low median 5-year survival of only 12.5% in the United States [9].

As such, there is an unmet need for the development of more effective strategies to treat patients with various subsets of CRC [10]. In the past decade, immunotherapy has elicited tremendous excitement owing to its success in achieving dramatic and durable responses in refractory solid tumors. High tumor mutation burden has emerged as a marker of responsiveness to immunotherapy in several tumor types, including melanoma and nonsmall cell lung cancer (NSCLC) [11, 12]. Current evidence suggests that some CRC tumors have high mutational load and can also respond to immunotherapy [13].

### 1.2. Colorectal Cancer Carcinogenesis- Genetic Pathways

Colorectal carcinogenesis is characterized by malignant transformation that involves the stepwise accumulation of multiple genetic alterations, thus favoring the proliferation and growth of neoplastic cells [14–16]. CRCs arise from two distinct molecular genetic pathways, the first involves chromosomal instability (CIN) and the second involves microsatellite instability (MSI) [17–20].

#### 1.2.1. Chromosomal Instability Pathway

The CIN pathway is responsible for the development of 75–80% of sporadic CRCs that are characterized by a high frequency of allelic imbalance, chromosomal amplifications, and translocations [14, 21, 22]. It results from a series of genetic alterations that involves the activation of proto-oncogenes such as K-RAS and the inactivation of tumor-suppressor genes, such as TP53, APC, SMAD2, and SMAD4 [14, 21–24].

#### 1.2.2. Microsatellite Instability Pathway

Mismatch Repair (MMR) is an essential mechanism that cells use to repair damaged deoxyribonucleic acid (DNA). It recognizes and repairs DNA base insertions, deletions, and mismatches that arise as a consequence of DNA polymerase slippage during replication [22, 25, 26]. Mutational or epigenetic silencing of the four most common MMR genes, MutL homolog 1 (MLH 1), MutS homolog (MSH) 2, MSH6, and postmeiotic segregation 2 (PMS 2), results in MSI [27–29]. This is characterized by markedly elevated rates of intragenic mutations of short, tandemly repeated DNA sequences known as microsatellites [30, 31].

Although criteria used to define MSI have evolved, they remain somewhat elusive. Nevertheless, there is a consensus that markers, including the mononucleotide repeats, BAT25, BAT26, and BAT40, and the dinucleotide repeats, D5S346, D2S123, and D17S250, are particularly useful for the identification of MSI tumors [32, 33].

#### 1.2.3. POLE Mutations

POLA1, POLD1, and POLE are polymerases encoded by the POLE gene, and they contribute to DNA repair and recombination processes [34, 35]. The role of POLE mutations in carcinogenesis has been demonstrated in CRCs and endometrial cancers [35]. These mutations were initially described in the Cancer Genome Atlas study [36]. Around 25% of hyper-mutated CRCs were found to have somatic POLE mutations and missense or nonsense MMR gene mutations in the absence of any MSI or MLH1 hyper-methylation. These were designated as ultra-mutated CRCs [36, 37].

Somatic POLE mutations can result in MSS ultra-mutated CRCs. Germline POLE and POLD1 mutations alter the exonuclease domains of POLE/POLD1 that are responsible for adequate proofreading. This culminates in an impaired MMR process during DNA replication. As such, carriers of germline POLE and POLD1 mutations tend to develop tumors that, despite being microsatellite stable (MSS), have an extremely high mutation frequency that can exceed a million base substitutions per genome [38]. Nevertheless, no strong correlation has been demonstrated between the increased mortality in MSS CRC patients and the presence of mutations in the proofreading domains of POLE/PODL1 [39].

Interestingly, MSS CRCs resulting from POLE mutations are distinct from the usual CRCs with an MSS phenotype in that the former tend to be ultra-mutated. Their high mutational load and increased expression of tumor-associated antigens render them potentially responsive to the anti-PD-1 antibody, Pembrolizumab [40].

### 1.3. Colorectal Cancer Categories Based on the Mismatch Repair Status

CRC tumors can be categorized into two discrete groups based on their mutation patterns and the proportion of markers showing MSI. Cancer cells with deficient MMR (dMMR) have mutation rates that are 100- to 1,000-fold as compared to normal cells [13, 41, 42]. Because mutations in these tumors are mostly seen at microsatellites, dMMR tumors have also been termed MSI-high (MSI-H) (hence the term dMMR-MSI-H) [43]. As a result of their high mutation burden, dMMR-MSI-H CRC tumors can present neoantigens on major histocompatibility complex (MHC) class I molecules, thus priming T-cells to recognize them as foreign. On the other hand, tumors that have a proficient MMR (pMMR) signature have a much lower mutation burden with a rate of less than 8.24 mutations per 106 DNA bases [44, 45]. Since only a minority of markers in these tumors show evidence of MSI, pMMR malignancies have also been termed MSI low (MSI-L) (hence the term pMMR-MSI-L).

Approximately 15% of patients with CRC have dMMR-MSI-H tumors [46, 47]. This rate decreases by stage, with approximately 5% of patients with mCRC demonstrating the dMMR-MSI-H phenotype [43]. In contrast, the remaining 95% of mCRC cases have the pMMR-MSI-L phenotype [48].

dMMR-MSI-H CRC tumors have a distinct pathologic phenotype when compared with other CRC phenotypes, including pMMR-MSI-L ones [49–51]. While the first subset of tumors more commonly originates in the proximal colon and is right-sided, the second subset is usually found at distal sites and is usually left-sided [30, 52, 53]. Also, unlike pMMR-MSI-L tumors, d-MMR-MSI-H tumors tend to be mucinous and poorly differentiated, with an expansile growth pattern, histologic heterogeneity, and increased tumor-infiltrating lymphocytes (TIL) [17, 53–56].

Interestingly, a significant relation was observed between the MSI status and patients' age. Results from several studies by Huang et al. [57], Yuan et al. [58], Jenkins et al. [59], and Greenson et al. [60] have noted that whereas patients above 50 years of age tend to have MSS CRC tumors, younger ones are more likely to have d-MMR-MSI-H ones. As such, most data suggest that an age under 50 years is a strong predictor of MSI.

### 1.4. Microsatellite Instability Status Identification Methods

Defective DNA MMR in CRC tumors can be detected by the lack of immuno-histochemical staining of the four MMR proteins, MLH1, MSH2, MSH6, or PMS2. It can also be identified using polymerase chain reaction (PCR) by testing for variation in the lengths of microsatellites between patients' tumor cells on one hand, and blood or normal tissue samples on the other. The latter method is based on consensus guidelines that recommend testing for the five specific microsatellites, BAT25, BAT26, D2S123, D5S346, and D17S250, and considering a tumor to be of dMMR-MSI-H phenotype when more than 30% of tested microsatellites have length variation compared to normal tissue [43]. In addition to the above-mentioned methods, the use of computational analyses of tumor next-generation sequencing has also provided an effective approach in the detection of MSI status in the last 5 years [61–63].

### 1.5. Indications for Microsatellite Instability Status Identification

Testing for MSI status in CRC tumors was initially used to identify patients in whom further germline testing for Lynch syndrome was warranted [64]. This is mainly because tumors in patients with Lynch syndrome were noted to more likely be of the dMMR-MSI-H phenotype. Also, around 33% of patients with dMMR-MSI-H CRC tumors were found to have Lynch syndrome or hereditary nonpolyposis CRC [65–69]. At present, it is recommended that all mCRC patients undergo testing for dMMR-MSI-H status in order to not only identify patients with Lynch syndrome, but also to guide therapy approaches, including treatment with anti-PD-1 blockade [43].

### 1.6. Prognostic Implications of the Different Colorectal Cancer Categories

It has been established that patients with dMMR-MSI-H CRC have superior overall survival (OS) outcomes and are less likely to develop distant metastases than those with pMMR-MSI-L CRC [52, 70]. This positive prognostic implication, however, is no longer valid in case a dMMR-MSI-H CRC tumor metastasizes or relapses following initial treatment. In fact, it has been shown that in the previous scenario, the prognosis might even become worse than that observed in pMMR-MSI-L mCRC [46, 71, 48].

Several studies have demonstrated an improved survival in patients with dMMR-MSI-H CRC tumors. For instance, in one population-based study that included 1,026 CRC patients, results showed a 60% drop in CRC-related deaths in patients with dMMR-MSI-H tumors. Interestingly, most of the risk reduction took place among stage III patients [72]. Similarly, in the Ontario population-based study that included 607 CRC patients, it was concluded that dMMR-MSI-H tumors appear to be predictive of a relatively favorable outcome [52].

When it comes to the relation between the MSI status of tumors and their potential to metastasize, studies indicate that regardless of the depth of tumor invasion, CRCs with dMMR-MSI-H phenotype are less likely to spread to regional lymph nodes or distant organs. This might explain why stage IV dMMR-MSI-H CRC constitutes only 2–4% of all mCRCs. In addition, significant results of a pooled analysis revealed that stage II pMMR-MSI-L tumors are 1.6 times more likely than stage II dMMR-MSI-H tumors to recur [73].

Despite promising data on the improved prognosis of patients with dMMR-MSI-H CRC, many conflicting reports exist. For instance, in a study reported by investigators from Scotland on patients with CRC, those who were 30 years of age or younger were mostly found to have mucinous tumors of the dMMR-MSI-H phenotype. While this cohort was found to have a relative risk of death of 0.87, patients who were older than 30 years of age had a lower relative risk of death of 0.11 [74].

## 2. Management: Systemic Therapies and Evolving Paradigms in Immunotherapy

Currently, the combination of cytotoxic and biologic agents is the standard treatment approach in mCRC. Several factors influence the treatment choice in patients with mCRC, including tumor and patient characteristics [75].

### 2.1. Systemic Therapies—The Predecessors of Immunotherapy

Compared to monotherapy, combinations of oxaliplatin or irinotecan and 5-fluorouracil (5-FU) or capecitabine have been shown to have higher response rates and improved progression-free survival (PFS) and OS. Several randomized-controlled trials have confirmed the efficacy of FOLFIRI, FOLFOX, FOLFIRINOX, CAPIRI, and CAPOX in mCRC, whether with or without vascular endothelial growth factor (VEGF) or epidermal growth factor receptor (EGFR) inhibitors [76–84]. In case cancer progression occurs while a patient is on an oxaliplatin-based regimen, an irinotecan-based regimen is recommended, and vice versa. In case of chemo-resistance, Regorafenib, a multi-tyrosine kinase inhibitor, or trifluridine, an antiviral drug, can be used in combination with tipiracil [85, 86]. However, outcomes from the use of these agents remain suboptimal [87].

### 2.2. Immune Checkpoint Blockade

The success of ICIs in an increasing range of heavily mutated tumor types such as melanoma has culminated in their exploration in different subsets of patients with mCRC. As a result of the dramatic and durable RRs obtained in patients with chemorefractory dMMR-MSI-H mCRC following treatment with ICIs, they have become potential alternatives to classical systemic therapies.

To date, the benefit of immunotherapy is mostly confined to a small subset of patients with dMMR CRC that only represents about 4–5% of patients with mCRC. Unfortunately, however, currently approved ICIs have been shown to be largely unsuccessful in patients with the pMMR-MSI-L phenotype that comprises 95% of all mCRC cases. This highlights the need to develop suitable novel treatment strategies for these patients. Current immunotherapeutic strategies being evaluated include combinations of ICIs with chemotherapy, VEGF inhibitors, cancer vaccines, adoptive cell transfer, or BTC antibodies [88, 89].

### 2.3. Rationale for Immunotherapy in Colorectal Cancer

Since ICIs were first reported in 2010 and 2012, they have translated into a significant OS advantage in comparison to established therapies in metastatic melanomas and NSCLC [90]. Given the background of chronic inflammation in the pathogenesis of most gastrointestinal cancers, the use of immune-based treatment approaches in them might have a role in releasing the brakes created by the tumor on the immune system and in harnessing an immune response to electively kill tumor cells [91, 92].

As previously mentioned, one of the leading causes of hypermutation in CRC is a defect in the DNA MMR system, which results in MSI-H tumors that strongly express various immunological checkpoint proteins, including cytotoxic T-lymphocyte associated antigen 4 (CTLA-4), PD-1, and programmed-death ligand 1 (PD-L1). These results in tumor escape from the host immune response by counteracting the active immune microenvironment of the MSI-H tumor and preventing the elimination of neoplastic cells.

CTLA-4 receptor is exclusively expressed on regulatory T-cells (T-regs), naive T-cells, and activated T-cells, and it acts as a regulator of immune cells [93–95]. Through its binding to CD80 and CD86 located on antigen presenting cells (APCs), it contributes to phosphatase activation and promotes the overall deactivation of T-regs [96–98].

PD-1 receptor belongs to the CD28 superfamily and is expressed on T-regs, B-cells, and myeloid-derived suppressor cells (MDSCs) [99]. The PD-L1/PD-1 axis induces T-cell exhaustion by transmitting coinhibitory signals and limiting tumor-infiltrating lymphocytes (TILs) and T-cell proliferation in peripheral tissues. This results in effective immune resistance in the tumor microenvironment [100, 101].

**Figure 1:**
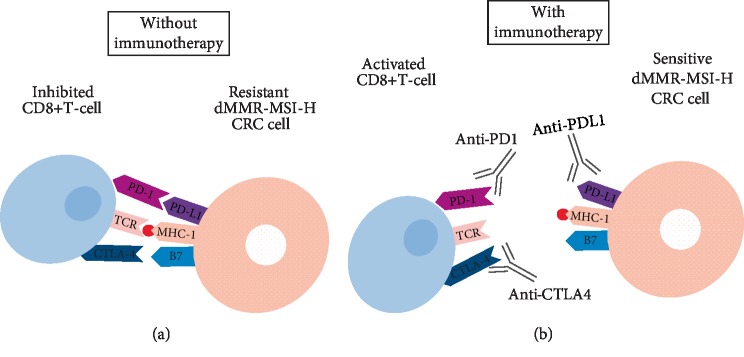
As a means to evade the immune-mediated killing, dMMR-MSI-H tumor cells tend to upregulate the expression of T-cell inhibitory ligands, including B7 (CD80, CD86) and PDL1, which bind to the co-inhibitory CTLA4 and PD1 receptors on immune cells, respectively. In order to overcome these limitations and to reduce the rate of tumor recurrence in this subset of CRCs, an immune-based treatment approach targeting CTLA4 and PD-1 or PD-L1 might be of help in harnessing an immune response to effectively kill tumor cells. As such, IICIs exploit the pre-existing inflamed microenvironment of dMMR-MSI-H CRC tumors to antagonize their T-cell inhibitor signals and result in their cytotoxic destruction.

In order to overcome limitations created by the PD-L1/PD-1 interaction and to reduce the rate of tumor recurrence in these tumors, an immune-based treatment approach targeting PD-1 and CTLA-4 on immune cells and PD-L1 on tumor cells may be beneficial [96, 102] (Figure 1). For instance, the use of anti-PD-1 therapy in patients with dMMR-MSI-H CRC tumors has been linked to significantly improved PFS and OS. Interestingly, the upregulation expression of PD-1, PD-L1, and CTLA4 in dMMR-MSI-H tumors that have metastasized might render this subset of patients more responsive to immune checkpoint blockade [48, 103].

In contrast, current ICIs have shown no clinically significant responses in patients with pMMR-MSI-L CRC tumors. This highlights the clinical significance of identifying the MSI status and hypermutated phenotype as a predictive marker for response to immuno-modulating agents.

### 2.4. Biomarkers of Response to Immune Checkpoint Blockade

Presence of dMMR-MSI-H in CRC tumors, as well as in other solid tumors, has been shown to be an effective biomarker in predicting durable and possibly curative responses to ICIs. However, the fact that not all patients with CRC respond well to ICIs highlights the need to identify more precise and reliable predictive biomarkers in evaluating response to immunotherapy.

#### 2.4.1. Mutational Burden and Neoantigen Density

Results from studies performed on patients with metastatic melanoma and NSCLC have shown durable responses and improved OS following treatment with ICIs [104–112]. Since these tumors were found to have high mutation burdens, they were thought to present more peptide neoantigens on their MHC class I molecules, thus being recognized as nonself and priming T-cells for activation and cytotoxic killing [113, 114]. This alteration in peptide sequences in these tumors was linked to the improved responses observed following treatment with Pembrolizumab [113, 115, 116]. As such, a correlation between mutational load and response to immunotherapy was described.

In CRC, the classic model of carcinogenesis is the adenoma carcinoma sequence [117–121]. Based on this scheme, malignant CRC cells have the capacity to accumulate a number of immunogenic mutations and become a potential target for immunotherapy, even though they might remain less immunogenic than melanoma or NSCLC [122]. Compared with pMMR-MSI-L CRC tumors, those with the dMMR-MSI-H phenotype have a nearly 20-times-higher mutation burden, enabling them to generate mutant neo-epitopes and trigger robust host anti-tumor immune responses [123–125]. In contrast, the lower tumor mutation burden in the pMMR-MSI-L tumors results in wild-type neoantigens and less overall immune stimulation, thus contributing to the limited or absent response to single-agent ICIs [43].

**Figure 2:**
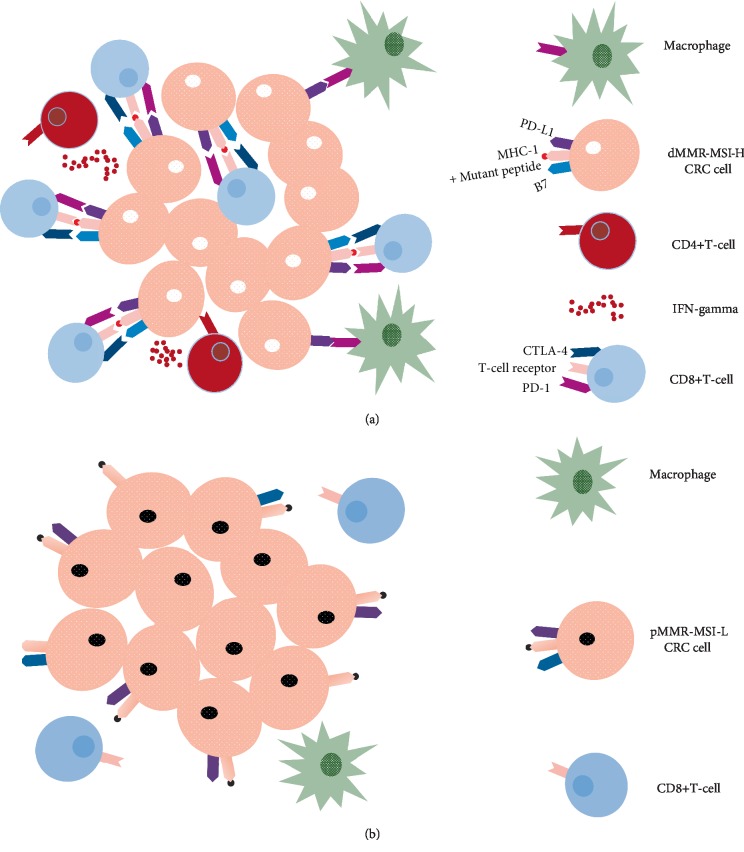
dMMR-MSI-H and pMMR-MSI-L CRCs have distinct tumor microenvironments. (a) dMMR-MSI-H tumor cells are characterized by their high rates of mutations that result in the presentation of mutated peptides on their MHC class I molecules. These are in turn recognized as foreign neoantigens by immune cells, resulting in high densities of cytotoxic CD8+ T-cell and T helper 1 CD4+ T-cell infiltration and elevated levels of IFN-gamma secretion. Tumor growth and progression is also influenced by the abundant tumor-associated macrophages present in the tumor microenvironment. As a means to evade the immune-mediated killing, dMMR-MSI-H tumor cells tend to upregulate the expression of T-cell inhibitory ligands, including B7 (CD80, CD86) and PDL1, which bind to the co-inhibitory CTLA4 and PD1 receptors. (b) By contrast, pMMR-MSI-L tumors generate wild-type peptides that are not immune-stimulatory and are thus characterized by much lower density of TILs.

Although mutational burden is an important predictive marker of potential response to immunotherapy, a high mutational load might not be sufficient to drive such a response. T-cell infiltration into the tumor bed is another marker that has also been associated with favorable outcomes [126–128]. For instance, there has been a longstanding awareness of the unique dMMR-MSI-H CRC tumor microenvironment. Histologic comparisons have revealed that it is being heavily infiltrated by CD8+ TILs, T helper 1 CD4+ TILs, and macrophages, [49, 51, 129]. In addition, immune responses generated against these tumors were described as Crohn's-like, especially with the accompanying elevation in neutrophil and platelet counts on the one hand and C-reactive protein and type I interferon (IFN) levels on the other [55, 56, 103, 126, 129–135]. This robust immune response is likely responsible for the favorable outcome of patients with primary resected dMMR CRC. In contrast the lower levels of TILs and the weaker immune response generated by tumors with pMMR-MSI-L tumors have been thought to be mechanisms of immune resistance and linked to the worse outcomes in this subset of patients [126, 136] (Figure 2).

On the basis of these data, further studies have used the approach of assigning an immunoscore based on the density and the location of tumor-infiltrating CD3+ CD8+ T-cells. This has been shown to be prognostic of improved clinical outcome in patients with early-stage CRC [126, 137, 138]. It has also culminated in reports of high immunoscores in patients with pMMR-MSI-L CRC. This raises the question of whether immune-phenotyping might enable prediction of immunotherapy response.

Hence, combining the microsatellite instability status with both, the immunogenic features of the tumor microenvironment and the mutational burden, might serve a better role in predicting response to immunotherapy.

#### 2.4.2. Beta 2-Microglobulin and Janus Kinase Mutations

Various biomarkers of response to anti-PD-1 therapy are currently being explored. While PD-L1 expression was found to be associated with ameliorated response and survival in patients with gastric cancer, gastro-esophageal junction cancer, and NSCLC [139, 140], this does not hold true in those with CRC [13, 141]. For instance, response to Nivolumab immunotherapy was shown to be independent from tumor PD-L1 expression in one study, with a similar percentage of responding patients seen in both, the PD-L1 positive and negative groups.

Although acquired mutations in Janus Kinase (JAK) 1, JAK2, and beta 2-microglobulin (B2M) are markers of resistance to PD-1 blockade in melanoma [142], their role in patients with CRC is not yet well defined. Truncating mutations in B2M result in impaired MHC class I antigen presentation and failure to elicit a T-cell response. Results from studies evaluating the use of Pembrolizumab in CRC tumors revealed that tumors developing resistance to this therapy had acquired B2M-mutations [136]. Surprisingly, patients with B2M-mutant dMMR-MSI-H CRCs have been shown to have a favorable prognosis [143]. As for inactivating mutations in JAK1 or JAK2, these have been also linked to resistance to anti-PD-1 therapy in melanoma [142, 144], but more research to evaluate their response to anti-PD-1 agents in CRC.

### 2.5. Current Therapies for Mismatch Repair-Deficient Microsatellite Instability-High Colorectal Cancer Tumors

#### 2.5.1. Single Agent Checkpoint Inhibitors

The tremendous survival benefit seen with ICIs among many patients with cancer has led to their widespread adoption in CRC [43]. ICIs initially demonstrated very limited clinical activity in nonselected CRC patients in early studies done between 2010 and 2013. For instance, in a study done on 45 patients with refractory CRC, treatment with Tremelimumab, an anti-CTLA4 immunoglobulin G2 antibody, resulted in a partial response (PR) in one individual whose MMR status was unknown [145].

On the basis of the knowledge of the immunogenic microenvironment of dMMR-MSI-H CRC tumors and the observed impressive tumor response, the interest in the use of immunotherapy in CRC grew, and several studies were initiated to explore the therapeutic potential of anti-PD-1 therapy in advanced CRC [13, 136, 146, 147].

In a study involving 19 patients with refractory cancers, treatment with the anti-PD-1 agent, Nivolumab, was initiated and no responses were initially reported [148]. Interestingly, however, one patient with dMMR-MSI-H CRC had a PR at 21 months and achieved a complete response (CR) that lasted more than three years after retreatment [149, 150].

In the open label phase II CheckMate 142 trial (NCT02060188), a cohort of 74 cases of dMMR-MSI-H mCRC was included and treated with Nivolumab monotherapy [146]. At a median follow-up duration of 12 months, an objective response (OR) was noted among 31% of patients, and disease control for 3 months or longer was observed in 69% of them. At the time the study was reported, all patients who responded to treatment were alive, and eight had responses lasting 12 months or longer with a Kaplan–Meier 12-month estimate of 86%. Response to immunotherapy was independent from tumor PD-L1 expression as a similar percentage of patients in the PD-L1 positive group (29%) and PD-L1 negative one (28%) responded to Nivolumab. Similarly, the presence of a history of Lynch syndrome or the BRAF or KRAS mutation did not seem to affect response to immunotherapy. The median PFS was 14.3 months, and the 12-month PFS was 50%. The 12 month OS was 73%.

When it comes to data on the safety of Nivolumab treatment, all-cause adverse events (AEs) were reported in 99% of patients, with 55% developing grades 3 and 4 events and 5% dying due to AEs that were not therapy-related. For instance, fatigue, diarrhea, pruritus, rash, and hypothyroidism were the most common grades 1 and 2 AEs, while pancreatitis, colitis, hepatitis and adrenal insufficiency were the most common grades 3 and 4 ones.

Based on the efficacy and safety results from the CheckMate 142 trial, the FDA has granted an accelerated approval for Nivolumab in July 2017 as a second line treatment option in adult and pediatric patients with dMMR-MSI-H mCRC that has progressed following treatment with a fluoropyrimidine, oxaliplatin, and irinotecan [146, 147, 151].

A similar positive effect in patients with dMMR chemorefractory mCRC was observed with the use of another anti-PD-1 antibody, Pembrolizumab. In the NCT01876511 phase II trial, cohorts of patients with dMMR-MSI-H CRC, pMMR-MSI-L CRC, and dMMR-MSI-H nonCRC were treated with Pembrolizumab [13]. Follow-up at 20 weeks revealed that 4 of the 10 patients with dMMR-MSI-H CRC had a PR and 5 had stable disease (SD). Updated results were presented during the 2016 American Society of Clinical Oncology (ASCO) Annual Meeting, and they described some response and disease control in 50% and 89% of the 28 patients with dMMR-MSI-H tumors, respectively. They also reflected no response in the 18 patients with pMMR-MSI-L CRC. In the dMMR-MSI-H cohorts, the 24 months PFS was 61%, and the OS was 66% [152]. In the pMMR-MSI-L cohort, however, the 5 months PFS was 2.2 months and the OS was 5.0 months. Response to immunotherapy was shown to be significantly correlated with the number of somatic mutations in the corresponding tumor [136]. Further analysis of this study confirmed the efficacy of Pembrolizumab in twelve types of dMMR tumors regardless of the tissue of origin [136].

In the phase I Keynote 016 clinical trial, the efficacy and safety of Pembrolizumab monotherapy were evaluated in 10, 9, and 18 patients with dMMR-MSI-H mCRC, dMMR-MSI-H nonCRC, and pMMR-MSI-L mCRC tumors [13, 136]. The initial report demonstrated that patients with dMMR-MSI-H mCRC had a 40% RR, while those with pMMR-MSI-L mCRC had a 0% RR. Updated results on 40 dMMR-MSI-H mCRC patients reported a RR of 52% and an estimated 2-year PFS rate of 53%, with higher somatic mutation loads being correlated with prolonged PFS. Updated results on 86 patients with dMMR-MSI-H tumors belonging to one of twelve different types such that the most common one was dMMR CRC (46.5%) were also reported. Patients with CRC had an overall RR of 52%, and those with dMMR nonCRC had an overall RR of 54%. A 53% objective radiographic RR and a 21% CR rate were also observed in a subgroup of patients with dMMR tumors [136]. 1- and 2-year PFS were estimated to be 64–53%, respectively. For instance, of the 18 patients who discontinued therapy at 2 years after being treated per protocol, none have had a recurrence at a median follow-up of approximately 8 months. These results suggest that in addition to its efficacy in patients with dMMR CRC, Pembrolizumab monotherapy seems to have durable responses that may result in cures.

When it comes to data on the safety of Pembrolizumab treatment, grade 3–4 or severe AEs were reported in 14% of patients in the Keynote 016 study. These included pancreatitis, colitis, thrombocytopenia, leukopenia, and anemia. Other less severe but major AEs were pruritus, arthralgia, thyroid dysfunction, anorexia, and fatigue.

Based on the efficacy and safety results from the Keynote 016 study, the FDA has granted an accelerated approval for Pembrolizumab in May 2017 as a second line treatment option in adult and pediatric patients with unresectable or metastatic dMMR-MSI-H solid tumors that have progressed on or after prior treatment with fluoropyrimidine, oxaliplatin, and irinotecan and who have no satisfactory alternative therapy options [151].

At present, a number of ongoing studies are evaluating the use of anti-PD-1 or anti-PD-L1 inhibitors in patients with dMMR-MSI-H mCRC. For instance, the efficacy of Pembrolizumab as a first-line treatment in stage IV dMMR-MSI-H CRC is currently being assessed in the phase III Keynote-177 trial (NCT02563002) with primary end points of PFS and OS, and a secondary end point of OR rate [153]. Also, the use of the anti-PDL1 antibodies, Atezolizumab, Avelumab, and Durvalumab, in the first-line metastatic setting is being explored by several clinical trials [10, 43].

#### 2.5.2. Combination of Chemotherapy and Immunotherapy or Anti-Angiogenic Agents

In order to improve outcomes in patients with mCRC, current strategies being investigated include combining one of the above-mentioned newly approved therapies with the current standard of care biologics and chemotherapy regimens such as FOLFOX, FOLFIRI, or FOLFIRINOX. By directly imparting damage to cancer cells and releasing antigens, cytotoxic agents could enhance the immune response against CRC tumors, thus further ameliorating the effects of immunotherapy. This approach, however, might be accompanied by cumulative AEs and negative interactions between systemic chemotherapy and immunotherapy.

This sheds light on the importance of initiating clinical studies that assess the safety and efficacy of combining immunotherapy with chemotherapy or biologics [10]. For instance, in the underway NCT02997228 trial, 347 CRC patients will be treated in the first-line setting with either Atezolizumab or with the combination of the first-line chemotherapy regimen, FOLFOX, plus the VEGF antagonist, Bevacizumab, or with the combination of both treatments. The primary trial end point is PFS, and secondary end points include OS and OR rate [154].

The randomized phase III NCT02912559 trial has been developed in an effort to determine the potential efficacy of Atezolizumab in combination with folinic acid, fluorouracil, and oxaliplatin as an adjuvant therapy in 700 patients with stage III dMMR-MSI-H CRC [43, 155, 156]. In the experimental arm, patients receive Atezolizumab plus FOLFOX for 6 months followed by Atezolizumab monotherapy for 6 months. Disease-free survival (DFS) and OS constitute the primary trial end points, and the incidence of AEs comprise its secondary end points.

#### 2.5.3. Combination of Immune Checkpoint Inhibitors

These results, suggesting additional clinical benefit with a combinatorial approach for patients with dMMR-MSI-H CRC tumors, have laid the groundwork for the exploration of additional combination therapies in this subset. Combining two immunotherapeutic agents is an alternative approach that might be of help in patients with mCRC. Results from a phase Ib trial suggested that the anti-PD-L1 antibody, Durvalumab, and the anti-CTLA-4 antibody, Tremelimumab, can be combined safely in this population with improved outcomes [157].

The phase II randomized trial, known as the CCTG CO.26 trial, was among the first studies to demonstrate that combined PD-L1 and CTLA-4 inhibition prolongs survival in patients with advanced refractory CRC. In this trial, 180 patients with advanced refractory CRC who were unselected for MSI were included between August 2016 and June 2017 [158]. They were then randomized in a 2 : 1 ratio to receive either best supportive care (BSC) alone or in addition to the immunotherapy combination of Durvalumab and Tremelimumab. Results revealed that patients in the combination group had significantly prolonged OS and preserved quality of life compared to those receiving only BSC. In fact, after a median follow-up of 15.2 months, patients receiving the immunotherapy combination had a median OS of 6.6 months, while patients receiving only BSC had a median OS of 4.1 months with a stratified hazard ratio (HR) of 0.72. Also, patients in the immunotherapy combination plus BSC arm and those in the BSC arm had a median PFS of 1.8 and 1.9 months, respectively, with an HR of 1.01 [157]. Interestingly, the OR for the disease control rate was 4.16 and was in favor of the immunotherapy combination arm. This was mainly reflected by the fact that the majority of patients in the first arm had SD and a higher disease control rate of 22.7% as compared to only 6.6% in the second arm.

When it comes to safety data on the combination of Durvalumab and Tremelimumab, results showed that AEs were more frequent in patients receiving this immunotherapy combination and included abdominal pain, fatigue, lymphocytosis, and eosinophilia. Whereas 64% of patients in the Durvalumab and Tremelimumab plus BSC group had an adverse event of grade 3 or higher, only 20% in those in the BSC group did [157]. When it comes to quality of life (QOL), however, patients receiving the immunotherapy combination had numerical advantage in terms of global health status and deterioration in physical function over those receiving only BSC based on the EORTC QLQ-30 questionnaire [157].

Promising preliminary results of an ongoing single arm trial evaluating the use of the combination of Nivolumab and the anti-CTLA-4 agent, Ipilimumab, in patients with resectable, early-stage CRC were presented at the 2018 European Society for Medical Oncology (ESMO) meeting [159]. Recruited patients were operated on after six weeks of signing the informed consent. Safety and feasibility constituted the primary trial end points, and pathological response comprised its secondary end point. Interestingly, a major pathological response was observed in all of the seven patients with dMMR-MSI-H tumors, with 57% of them having CRs. Also, significant upregulation in TILs was noted in patients with dMMR-MSI-H with a significant *p*-value of 0.0009. These results raise a controversial question of whether curative surgical resection, which has long been the mainstay of treatment in patients with early-stage CRC, might be safely evaded in a subset of dMMR-MSI-H CRC.

In the previously mentioned CheckMate 142 trial that included a single-agent Nivolumab cohort, the efficacy and safety of combining Nivolumab with Ipilimumab were also explored in a cohort of 119 previously untreated patients with stage 4 dMMR-MSI-H CRC [147]. Results on the first 45 recruited patients were reported at the 2018 ESMO Annual Meeting [160]. After a median follow-up of 13.8 months, the objective RR was 60%, the disease control rate was 84%, and the CR rate was 7%. At 12 months, the PFS and OS values were 77% and 83%, respectively [160].

Updated results on the complete cohort of 119 patients receiving the immunotherapy combination further demonstrated improved outcomes with this treatment plan over Nivolumab monotherapy. For instance, after a median follow-up duration of 13.4 months, the OR rate and the tumor burden reduction from baseline were seen in 55% and 77% of patients, respectively [147, 161]. Among the 58 patients who responded, 5 had CRs and 53 had PRs. Also, the response in the majority of responders (83%) lasted for 6 months or longer. Interestingly, an analysis on a subgroup of 82 patients who had progressed on a fluoropyrimidine-, oxaliplatin-, and irinotecan-containing regimen yielded an OR rate of 46%. This is the setting in which the combination of Nivolumab and Ipilimumab is approved [147]. Results also showed improvement in survival, with a 9-month and 12-month PFS of 76% and 71%, respectively, and a 9-month and 12-month OS of 87% and 85%, respectively.

As a result of these results suggesting additional clinical benefit and durable responses with Nivolumab and Ipilimumab, the FDA granted its approval to this combinatory approach in patients with refractory CRC (rCRC) that has progressed on a fluoropyrimidine-, oxaliplatin-, and irinotecan-containing regimen in July 2018 [151].

Although the combination of anti-PD-1 and anti-CTLA-4 agents proved to be a synergistic combination and showed promise over anti-PD-1 monotherapy, it resulted in an increased rate of major treatment-related adverse effects. For instance, 73% of patients in the combination arm reported any-grade AEs including diarrhea (22%), fatigue (18%), pruritus (17%), fever (17%), hypothyroidism (13%), hyperthyroidism (11%), and hepatitis (7%). While 20% of patients who received Nivolumab monotherapy experienced grades 3–4 AEs, 32% of those treated with Nivolumab and Ipilimumab combination developed such events. These mainly included hepatitis (11%), pancreatitis (4%), anemia (3%), and colitis (3%) [147].

Despite the fact that single and dual checkpoint blockade were more effective for dMMR-mCRC than chemotherapy, ambiguity remains concerning their economic impact. According to the decision analytic model by Chu et al., ICIs were not found to be cost-effective when compared with chemotherapeutic agents, largely because of their elevated drug costs [162].

In spite of the compelling data in dMMR-MSI-H CRCs, to date, no drug or combination has been granted approval by the European Medicines Agency (EMA), pending results of ongoing trials (Tables 1 and 2).

### 2.6. Response of Mismatch Repair-Proficient Microsatellite Instability-Low Colorectal Cancer Tumors to Current Immunotherapeutic Agents

Unlike in patients with dMMR-MSI-H CRC, single agent immunotherapy has demonstrated limited or no clinical benefit in patients with pMMR-MSI-L CRC, who comprise around 95% of patients with mCRC [163]. In the pivotal Pembrolizumab study, no response was observed in patients with pMMR-MSI-L disease [13].

In the Check Mate 142 study, limited responses were seen in pMMR-MSI-L tumors, with one of 20 patients responding to the combination of PD-1 and CTLA4 blockade [147]. Similarly, in the ongoing single-arm trial on patients with resectable, early-stage CRC treated with the combination of Nivolumab and Ipilimumab that was presented at the 2018 ESMO meeting, no major pathological responses were noted among the 8 patients with pMMR-MSI-L tumors although significant increases in their TILs was noted [164].

### 2.7. Potential Strategies that Might Render Mismatch Repair Colorectal Cancers More Responsive to Immunotherapy

The challenging process of developing suitable immunologic treatment approaches for patients with pMMR-MSI-L CRC is highlighted by the stark contrast in rates of disease control and tumor regression between patients with dMMR-MSI-H CRC on one hand, and those with pMMR-MSI-L CRC on the other hand following treatment with anti-PD-1 therapy. As such, understanding differences in tumor molecular patterns, immune cell content, and cytokine expression that render dMMR-MSI-H tumors more responsive to treatment is of utmost importance. Doing so, for instance, would enable the replication of favorable immune manipulations within pMMR-MSI-L CRC tumors, hence rendering them more like dMMR-MSI-H CRC tumors [43].

Gene expression profiling has recently demonstrated significant differences between both categories of tumors in terms of their effects on the immune system. For instance, in their comparison of the genes expressed in primary dMMR-MSI-H and pMMR-MSI-L CRC tumors, Mlecnik et al. noted that the majority of the differentially regulated ones were descriptive of CD8+ cytotoxic and CD4+ T-helper 1 cell types [137, 156].

As mentioned before, compared to patients with pMMR-MSI-L CRC, those with dMMR-MSI-H CRC had higher levels of immuno-stimulatory cytokines and chemokines including IFN-gamma, interleukin (IL) 15, CCL3, and CXCL16 and lower levels of the monocyte chemotactic agent, CXCL14. Also, while the high degree of intestinal anti-tumor T-cell infiltration in dMMR-MSI-H CRC has been linked to improved activity of PD-1/PD-L1 blockade [96], the greater extent of immuno-inhibitory T-regs and MDSCs infiltration within pMMR-MSI-L tumors may explain their resulting poor immune response [166].

As previously mentioned, one genomic explanation for this limited ability to recruit anti-tumor immune cells in pMMR-MSI-L mCRC tumors is related to their low mutational burden that is responsible for their low frequency of neoantigens [166]. In fact, the neoantigen load has been linked to the extent of TILs in CRC tumors including those that are of dMMR-MSI-H and pMMR-MSI-L phenotype [167]. Since the cutoff corresponding to the neoantigen load associated with increased T-cell infiltration is well above that seen in most pMMR-MSI-L, the need to develop strategies that will target an immune response against the antigens displayed by the malignancy and enhance T-cell infiltration in pMMR tumors with lower mutational burden is essential [166].

This has been supported by other studies showing a correlation between the extent of TILs and improved outcomes in pMMR-MSI-L CRC patients similar to that seen in dMMR-MSI-H CRC ones [126, 168]. Interestingly, in the analysis done by Mlecnik et al., pMMR-MSI-L CRC malignancies were further categorized into a first group that was relatively similar to dMMR-MSI-H CRC tumors in terms of both, the levels of T-helper 1 cells, cytokines, and chemokines and the extent of cytotoxic gene expression, and a second group that lacked these profiles. Results showed that the prognosis of patients in the first group was similar to that observed with dMMR-MSI-H tumors and was notably better than that seen in the second group.

Although pMMR CRC tumors are characterized by their lower mutational and neoantigen burden, they express cancer germline antigens and harbor mutated KRAS or p53 proteins against which T-cell responses have been identified [169, 170]. In addition, isolation of T-cells that recognize pMMR CRC tumor antigens have been reported [171]. No matter how much low the frequency of TILs in the pMMR CRC tumor microenvironment is prior to any therapy, their presence provides some insight concerning the role of the cancer immunity cycle activation phase to prime antigen-specific CD4+ and CD8+ T-cells.

Therefore, it is possible for pMMR CRC to have a dMMR-like immune phenotype; however, whether these dMMR-like pMMR CRCs will experience responses to checkpoint blockade or any other immunotherapy that mirrors the clinical responses of dMMR CRC is unknown [43]. As a result, there has been considerable interest in combining immune checkpoint blockade with other immune-modulating agents in an attempt to potentially increase the efficacy of immunotherapy in pMMR-MSI-L tumors by further promoting T-cell accumulation in tumors, limiting T-cell exhaustion, and improving tumor immune recognition by increasing MHC class I expression. Despite promising results in an early phase I trial, a confirmatory phase III trial evaluating the use of the anti-PD-L1 agent, Atezolizumab, with and without the mitogen activated protein kinase (MEK) 1/2 inhibitor, Cobimetinib, failed to replicate the benefit over the multi-tyrosine kinase inhibitor, Regorafenib, in unselected patients with chemotherapy refractory pMMR-MSI-L mCRC [172].

The remainder of this section will focus on combination strategies under development to enhance effector T-cell activation and infiltration into CRCs and decrease the immune-inhibitory cell population (Tables 3 and 4).

#### 2.7.1. Radiotherapy, Chemotherapy, or Antiangiogenic Agents

Radiotherapy (RT), chemotherapy, and anti-angiogenic agents are current standard therapies that may enhance immune activation by destroying tumor cells and releasing TAAs in the process. As a result, combining them with checkpoint blockade and other immunotherapies may play an essential role in improving outcomes in pMMR-MSI-L mCRC tumors.

Since RT causes DNA damage and primes T-cells through generating an enlarged neoantigen repertoire and since its abscopal effects in metastatic cancers have been repeatedly reported, it has become an active area of investigation [173, 174]. In this respect, the NCT02437071 trial was initiated to evaluate the use of Pembrolizumab in combination with either radiofrequency ablation (RFA) or external beam radiation therapy (EBRT) in patients with CRC [175]. Interim results were reported in 2016, and they showed that none of patients receiving RFA and one of the 22 patients receiving EBRT had some response. The combination of RT and dual immune checkpoint blockade of CTLA4 and PDL1 has been linked to improved outcomes and tumor regression in patients with melanoma [176]. In the ongoing NCT03122509 trial, the efficacy of combining RT or radiofrequency ablation with dual immune checkpoint blockade of CTLA4 and PDL1 is being investigated [177].

Chemotherapy also induces immunogenic cell death and activates tumor-associated dendritic cells (DCs) and effector lymphocytes [178]. Several preclinical studies on lung cancer models have demonstrated the role of chemotherapy in sensitizing tumors to checkpoint blockade, and this supports the initiation of studies to evaluate this combination in other malignancies, including mCRC [179]. For instance, many ongoing studies are currently investigating the use of chemotherapy in combination with either anti-PD-1, anti-PD-L1, EBRT, or RFA in mCRC [43].

Other standard of care therapies in CRC include anti-angiogenic agents that might also demonstrate synergy when combined with immune therapies. For instance, Cetuximab, an anti-EGFR antibody, can activate antibody-dependent cellular cytotoxicity and Bevacizumab, an anti-VEGF antibody, can reduce the immuno-inhibitory effects of VEGF by reducing its free levels [180]. As such, several ongoing studies are currently assessing the immunomodulatory potential of combining anti-angiogenic agents with immunotherapy and chemotherapy, and preliminary results are promising.****In a trial on metastatic melanoma patients being treated with the either Ipilimumab alone or a combination of Bevacizumab and Ipilimumab, the addition of Bevacizumab in the second arm resulted in a remarkable increase in tumor-infiltrating CD8+ T-cell as compared to the first arm [181]. Such promising results were also reported in CRC. In the ongoing NCT01633970 phase Ib trial that investigated the combination of Atezolizumab plus Bevacizumab with or without FOLFOX in patients with refractory pMMR-MSI-L CRC tumors, preliminary results on 14 patients showed that 7% of them had ORs and 64% of them had SD [182, 183]. Further correlative analysis reflected some upregulation in CD8+ T-cell infiltration and PDL1 expression in mCRC tumors following chemotherapy administration with or without Atezolizumab and Bevacizumab [184].

#### 2.7.2. Targeted Therapy

The RAS–MAPK pathway plays an important signaling role in CRC and its activation has been linked to direct pro-proliferative effects on tumor cells and to decreased levels of tumor-infiltrating T-cells. As such, the effect of inhibiting MEK, a downstream effector of this pathway, was evaluated in several preclinical models. Results of these studies showed that Cobimetinib, a MEK inhibitor, induces IFN-gamma-dependent MHC1 and PD-L1 upregulation on tumor cells, thus augmenting anti-tumor activity by enhancing intra-tumoral CD8+ T-lymphocyte activation and synergizing with immune checkpoint blockade [185, 186]. As a result, the inhibition of the MEK-dependent intracellular signaling pathway was viewed as an effective approach in sensitizing pMMR-MSI-L mCRC for immunotherapy.

These promising results with the use of synergistic combination led to the initiation of the NCT01988896 phase Ib dose-escalation and dose-expansion trial that included a cohort of patients with refractory KRAS-mutant CRC, including pMMR-MSI-L CRC but no dMMR-MSI-H CRC, who were treated with the combination of Cobimetinib and Atezolizumab [187]. Escalating doses of the Cobimetinib (20 mg, 40 mg, and 60 mg) were administered on a daily basis for 21 days on, 7 days off, and an 800 mg dose of intravenous Atezolizumab was given every two weeks. Preliminary results were reported on 23 recruited patients in 2016 and revealed an overall RR of 17%, with four patients developing a PR with at least 30% decrease in tumor size and five patients having a SD. These responses ranged from 4 months to more than 15 months in duration, and they were still ongoing in two out of the four partial responders. The baseline PD-L1 status did not appear to affect response. Interestingly, however, three of the four responding patients had confirmed pMMR-MSI-L CRC, and the survival was as high as 13 months among those with confirmed pMMR CRC. No serious therapy-related AEs were reported at this stage of analysis in any patient.

At the 2018 ASCO Gastrointestinal Cancers Symposium, updated results on 84 included patients were presented and showed an overall RR of 8% and a disease control rate of 31%. These responses were durable, with a median duration of response of 14.3 months. Of note, 4 of the 7 responders had MSS and 1 had MSI-low mCRC, while the remaining 2 had unknown MSI status [163, 188]. Among all patients, the 6-month PFS and the 12-month OS rates were 18% and 43%, respectively. When only the subset of patients with MSS disease were considered, the 6-month PFS and the 12-month OS rates were decreased to 27% and 51%, respectively. These survival data are promising, especially that the 12-month OS rate obtained with the current standard of care multi-tyrosine kinase inhibitor, Regorafenib, is 24%.

As for safety data, the combination therapy was associated with a manageable AE profile, with most of the reported AEs being secondary to Cobimetinib. For instance, the most common treatment-related grades 3-4 AEs reported were rash, diarrhea, fatigue, and increased blood creatine phosphokinase. On the basis of data from this phase Ib trial, the combination of anti-PD-L1 immunotherapy and an MEK inhibitor represents the first potential immune-modifying therapy that might increase response to immunotherapy in patients with MSS mCRC who comprise 95% of CRC patients [189, 190].

These promising results culminated in the initiation of the phase III NCT02788279 IMblaze 370 (COTEZO) randomized trial that compares the efficacy of the above-mentioned combination therapy versus Atezolizumab monotherapy with the current standard of care treatment, Regorafenib monotherapy, in pretreated patients with refractory CRC [191]. Of note, 95% of recruited patients had pMMR-MSI-L tumors, while the remaining 5% had dMMR-MSI-H ones. Unfortunately, results reported at the ESMO 18^th^ World Congress on Gastrointestinal Cancer showed that the study failed to meet its primary endpoint and demonstrated that unlike Regorafenib monotherapy, both, the Atezolizumab and Cobimetinib combination therapy and the Atezolizumab monotherapy, did not result in a statistically significant OS [172].

Several ongoing studies are currently investigating the combination of immunotherapy and targeted therapy in patients with pMMR-MSI-L CRC. These include the phase Ib NCT02876224 trial evaluating the Cobimetinib, Atezolizumab and Bevacizumab combination and the phase II NCT02060188 study of combined Cobimetinib, Nivolumab, and Ipilimumab treatment [192, 193]. Also, the combination of MEK inhibition with PD-1-blockade and chemotherapy is currently being assessed by several trials.

In addition, combinations of other MEK and checkpoint inhibitors are under evaluation [188]. For instance, loss of PTEN and PIK3CA mutations is common in CRC, and this activates the PI3K pathway that in turn is linked to checkpoint upregulation [194]. As a result, combining the PI3K pathway blockade with checkpoint blockade might also have promising results and is currently under study.

#### 2.7.3. Tumor Vaccines

By supplying tumor antigens, tumor vaccines constitute an active form of immunotherapy that activates a host's immune response against cancer. Various types of tumor vaccines have been studies in patients with mCRC, and these include autologous and peptide vaccines, DC transplants, and oncolytic viral vectors encoding tumor antigens [195].

(a) *Autologous Vaccines*. Autologous vaccines contain overexpressed or mutated tumor-associated antigens (TAAs) from cancer patient as a means to trigger host T-cell responses against them [196]. These vaccines have been mostly evaluated in the preventive setting in CRC and have sometimes been modified by a nonlytic strain of the Newcastle disease virus [197, 198]. Since minimal benefit has been reported, this approach is still not used in clinical practice.
(b) *Peptide Vaccines*. Peptide vaccines contain antigenic epitopes derived from TAAs, with the most commonly targeted peptides being carcinoembryonic antigen (CEA), EGFR, and mucin 1 [199]. In a phase II trial, only a limited benefit was observed when 96 patients with chemotherapy-resistant mCRC were treated with an oxaliplatin-based chemotherapy regimen combined with a vaccine containing 5 human leukocyte antigen (HLA)-A^∗^2402-restricted peptides of which 3 were from oncoantigens and 2 were from VEGF receptors [200].
(c) *Dendritic Cell Transplants*. The typical approach in tumor vaccination includes autologous transplantation of DCs which are potent TAA-presenting cells in association with MHC class I and trigger T-cell immunity. In a randomized phase II clinical trial, an autologous tumor lysate DC vaccine was compared to the BSC [201]. Although results showed a tumor-specific immune response in the vaccinated arm, they failed to demonstrate improved disease control or survival as compared to the BSC arm. Nevertheless, evidence that supports the safe use of DC transplants in patients with advanced malignancies exists [202].
(d) *Oncolytic Viral Vector Vaccines*. The intra-tumoral or intravenous administration of competent oncolytic viruses constitutes another approach that generates a stronger immune response against tumor antigens than do peptide vaccines. Oncolytic viral vector vaccines are modified anticancer viruses that express immunomodulatory genes and make use of a viral vector system to deliver TAAs. They are capable of selectively infecting and lysing malignant cells without damaging normal tissue. During this process, TAAs are released and create an inflammatory milieu that enables innate immune responders such as DCs to process and present antigens to T-cells.

Although oncolytic virus therapy has proven to be efficacious in solid tumors such as melanoma, limited data concerning their efficacy in mCRC exists. Early studies evaluated the use of oncolytic Newcastle disease virus, Ad11/Ad3 chimeric group B adenovirus, reovirus, and herpes simplex virus in patients with CRC, and preliminary results were somewhat promising [203].

Positive safety and efficacy outcomes were also obtained from a phase I/II study that investigated the use of the genetically engineered oncolytic herpes simplex virus, NV1020, in patients with previously treated mCRC [204]. Results revealed that disease control was observed in 67% of recruited patients and that the 1-year survival rate was 47.2%.

In contrast, results from a randomized phase II clinical trial in which the nonreplicating canary pox virus expressing CEA and B lymphocyte antigen B7 (ALVACCEA/B7-1) vaccine was combined with the irinotecan-based chemotherapy in the treatment of 118 patients with mCRC showed no improvement in clinical or immune responses [89].

As a result of these conflicting results, oncolytic therapies remain experimental and have not become part of clinical practice to date [10, 205, 206]. The modest activity obtained with cancer vaccine monotherapy in the setting of minimal disease has resulted in several attempts to include them as part of combination therapies in an attempt to increase tumor-infiltrating CD8+ T-cells and decrease T-regs [207, 208].

Several ongoing trials are currently combining oncolytic viruses, peptide vaccines, or DC antigens with chemotherapy or ICIs with the aim of ameliorating tumor immunogenicity in the adjuvant and metastatic settings in CRC and other solid tumors.

#### 2.7.4. Adoptive Cell Transfer

Adoptive cell therapy (ACT) is an emerging modality for treating CRC. In this approach, cytotoxic T-cells are collected from patients' tumors, lymph nodes, or peripheral blood, and they are then infused into their blood stream with the goal of recognizing and destroying tumor cells and achieving a sustained response [209, 210].

Natural Killer (NK) cells obtained from umbilical cords of preclinical mouse models demonstrated positive outcomes in RAS and BRAF mutated neoplasms as well as Cetuximab-resistant ones [211, 212]. Promising results were also obtained in clinical studies on human subjects, whereby IL-2 or IL-15 incubated NK cell transplants have been proven to have some benefit in patients with refractory mCRC and a mutated EGFR status [213]. This approach is still under development and remains a young branch of immunotherapy.

Chimeric antigen receptors (CAR) T-cell immunotherapy constitutes T-cells that are engineered to express immuno-stimulatory ligands including lipid nanoparticles containing the IL-15, IL-12, or IL-7 receptor [214, 215]. By selectively binding to cancer cells, CAR T-cells enhance the tumor cells killing process. The efficacy of this therapy has been demonstrated in preclinical mouse models of mCRC [216]. Several groups have investigated the overly expressed marker in CRC, CEA, as a target for ACT [217–219]. In a small study, CAR T-cells targeting CEA were administered to three patients with mCRC [217]. One of the patients had an OR in lung and liver metastases, and serum CEA levels declined in all of them. However, all three patients developed severe colitis as a dose-limiting toxic effect. In another study, 7 of 10 patients with heavily treated mCRC had SD 4 weeks after CAR T-cell infusion, and 2 patients experienced tumor shrinkage [218]. At 30 weeks, 2 patients continued to have SD. In this study, treatment was well tolerated, with no reports of colitis.

Despite the fact that CAR T-cell therapy has been successfully used in the treatment of B cell malignancies [220, 221], its applicability in solid tumors, such as CRC, remains undetermined [222, 223]. The affordability and feasibility of such highly sophisticated cell manipulation approach present a major challenge.

#### 2.7.5. Bispecific T-Cell Engaging Antibody Therapy

Other strategies to target TAAs include bispecific T-cell- (BTC-) engaging antibodies that are a new class of engineered agents that simultaneously binds T-cells and tumor cells. This results in a large number of T-cells capable of recognizing and attacking tumor cells.

Preclinical data support the combination of ICIs and BTC–engaging antibodies targeting CEA antigen on tumors and CD3 on T-cells [224]. As such, this CEA-BTC antibody, also known as RG7802 or RO6958688, was evaluated in CRC [139]. For instance, in a study that assessed the use of the CEA-BTC antibody, alone or in combination with Atezolizumab, in patients with CEA positive solid tumors, 45% of the 31 patients with mCRC who received monotherapy had either a PR or SD [88]. 36% of the 25 who were treated with the combination therapy showed either a PR or SD.

Preliminary results from an ongoing phase I study that is investigating the use of the CEA-BTC antibody, alone or in combination with Atezolizumab, demonstrated upregulation in TILs and improvement in clinical responses in the combination therapy arm [225]. Nevertheless, patients in this arm experienced more adverse effects, with higher rates of infusion reactions, fever, and diarrhea. Two other ongoing phase I trials are currently exploring the use of CEA-BTC as a monotherapy (NCT02324257) [226] and in combination with Atezolizumab (NCT02650713) in patients with metastatic MSS CRC [227]. Encouraging results were reported in March 2017, whereby an improvement in clinical activity was observed in patients receiving the CEA-BTC monotherapy on the one hand, and a further enhancement in activity was noted when patients received the combination therapy with Atezolizumab on the other hand [228]. Of note, patients in the combination therapy arm had an overall disease control rate of 82% and an overall RR of 18%, with 64% of them having a SD. Overall, toxic effects were manageable. As such, CEA-BTC is among the first BTC antibodies to show efficacy in solid tumors in general and MSS CRC in particular [228].

#### 2.7.6. Indoleamine 2,3-Dioxygenase 1 Inhibitors

The enzyme Indoleamine 2,3-dioxygenase 1 (IDO1) is overexpressed by cancer cells and DCs and metabolizes tryptophan into the metabolite kynurenine [229]. T-cells will be deprived of tryptophan and suppressed by the effect of kynurenine on DCs [230]. In this way, IDO1 enzyme suppresses the immune response against cancer cells.

Blocking this immunosuppressive effect of IDO1 was thought to improve outcomes in cancer patients. Following the success of PD-1 and CTLA-4 checkpoint inhibitors in melanoma, attention has turned to other checkpoint proteins such as IDO1. Several early phase trials have evaluated the use of IDO1 inhibitors such as Epacadostat, Indoximod, and BMS-986205 [231], but results failed to reflect their efficacy as monotherapies. For instance, in a phase I trial in which 52 patients with various metastatic solid tumors were treated with Epacadostat no objective tumor responses were observed [232].

The main clinical interest of IDO1 inhibitors has been in combination with anti-PD-1 agents [233], especially that preclinical *in vivo* studies demonstrated significant synergy and increased tumor shrinkage with this combination as compared to anti-PD-1 monotherapy. The phase III ECHO-301/KEYNOTE-252 trial randomized 706 patients with unresectable or metastatic melanoma patients to either Epacadostat and Pembrolizumab or Pembrolizumab and placebo [234]. Results revealed no difference in PFS and OS rates at 12 months (37% and 74% in both arms, respectively). Also, the OR rate in the combination arm was very similar to that obtained with Pembrolizumab alone (34.2% and 31.5%, respectively). In addition, 21.8% of patients with the combination and 17% with Pembrolizumab alone encountered grade 3 or higher treatment-related AEs. Since OS was not expected to reach statistical significance and since the trial did not meet its primary endpoint of PFS, the external data monitoring committee agreed to discontinue the study.

Several ongoing trials are evaluating the use of the IDO1 inhibitors, Indoximod and Epacadostat in combination with ICIs including Pembrolizumab in various solid malignancies including pMMR-MSI-L mCRC [235].

#### 2.7.7. Depleting T-Regs and MDSCs

As a result of the high infiltration of pMMR-MSI-L CRC tumors with MDSCs and T-regs, it was thought that one way of making these tumors more responsive to immunotherapy could be achieved by depleting these cell types.

Results from preclinical studies on CRC mouse models showed that the use of an anti-CSF1R antibody could delay tumor growth by decreasing levels of tumor-infiltrating MDSCs. Similarly, other preclinical models have demonstrated synergy between anti-CSF1R antibodies and ICIs [236, 237]. Ongoing trials are currently exploring the use of combination of Nivolumab and the anti-CSF1R antibody, Cabiralizumab, in solid tumors on the one hand, and the combinations of Durvalumab and the anti-CSF1R antibody, Pexidartinib, in CRC [238].

Also, the observation that the anti-CCR4 antibody, Mogamulizumab, depletes CCR4 positive inhibitory T-regs in patients with cancer has paved the way for its evaluation in combination with other immune therapies for malignancies that are highly infiltrated with T-regs, including pMMR-MSI-L CRCs [239].

Regorafenib is a potent inhibitor of angiogenic and oncogenic kinases that has been shown to decrease the density of tumor-associated macrophages in murine models [240]. The fact that combining it with anti-PD-1/PD-L1 agents has further ameliorated its tumor growth suppression effects compared to either treatment alone has set the foundation for the NCT 03406871 trial [241, 242]. This ongoing trial aims at assessing the safety and efficacy of the combination of Regorafenib and Nivolumab in patients with previously treated, advanced gastric cancer or CRC. 25 gastric cancer and 25 CRC patients were recruited until October 2018, and they were exposed to both, Regorafenib (dose-escalation: 80–160 mg; frequency: once-daily at a 21 days on/7 days off schedule) and Nivolumab (dose: 3 mg/kg; frequency: once every 2 weeks).

Promising preliminary results were presented at the 2019 ESMO and ASCO meetings. Interestingly, 18 out of the 19 patients with an objective tumor response had MSS tumors (7 MSS CRC, 11 MSS gastric cancer, and 1 MSI-H CRC). Also, of the 7 gastric cancer patients who were pretreated with an anti-PD1 agent, 3 attained a partial response. In addition, a significant drop in the density of T-regs in pre- and post-treatment tumor samples was noted at tumor response. Consequently, it was concluded that combining Nivolumab with 80 mg of Regorafenib is safe and has an adequate anti-tumor activity in MSS CRCs and gastric cancers. Nevertheless, investigations in larger cohorts are warranted to better evaluate this combination therapy in MSS CRC patients.

In addition, adenosine that is generated from adenosine triphosphate in the tumor microenvironment results in immunosuppressive effects after binding to the A2A adenosine receptor expressed by immune cells. These include the inhibition of the proliferation of natural killer T-cells and the enhancement of the proliferation of T-regs and MDSCs [243–245]. Results from preclinical studies on murine CRC models have shown activity with the use of an anti-CD73 antibody alone or in combination with checkpoint blockade [245]. This led to the initiation of several trials evaluating the use of the anti-CD73 antibody, MEDI-9447, in combination with Durvalumab, in patients with different types of cancers, mainly lung cancer.

#### 2.7.8. Epigenetic Modulators

It has been noted that tumors exposed to an immune attack can downregulate tumor antigens, immuno-stimulatory IFNs, and MHC proteins. One proposed mechanism was through epigenetic regulation. As such, epigenetic modulators were developed to upregulate the above-mentioned immunomodulatory pathways and synergize with standard immunotherapies [246, 247]. Ongoing clinical trials on CRC are exploring different combinations of epigenetic modifiers and ICIs.

## 3. Conclusion

ICIs currently constitute the main domain of immunotherapy in patients with an increasing range of malignancies [10]. Their success in heavily mutated tumor types such as melanoma has culminated in their exploration in different subsets of patients with mCRC. Through boosting the host's immune response against tumor cells with limited collateral damage and through their dramatic and durable RRs among patients with chemorefractory dMMR-MSI-H mCRC, ICIs have become potential alternatives to classical systemic therapies. In this respect, the two anti-PD-1 agents, Pembrolizumab and Nivolumab, have been granted FDA approval for this subset of patients [248]. Unfortunately, however, since not all CRC cases with the dMMR-MSI-H phenotype respond well to ICIs, further studies are ongoing to better understand both, the mechanisms that render some of these tumors resistant to immunotherapy and the biomarkers that provide them with positive prognostic implications.

Another challenge lies in developing suitable novel treatment strategies for the other subset of patients with the pMMR-MSI-L phenotype that comprises 95% of all metastatic CRC cases. Many questions remain with regard to the optimal way to harness immunotherapy in this subset, especially that no response to single agent checkpoint inhibitors was noted in most studies. As such, more research is required to further understand the potential mechanisms that make most pMMR tumors resistant to current immunotherapies. In addition, there is a need for further insight into the best strategies that would alter the tumor environment in pMMR-MSI-L tumors in a way that would render it similar to that of dMMR-MSI-H ones, thus rendering them potentially more responsive to current immunotherapy regimens.

With the progress in several scientific and medical fields and the growing surge in knowledge about CRC and its tumor microenvironments, new pharmacological strategies are being developed in an attempt to improve outcomes in patients with pMMR-MSI-L CRC tumors. Current approaches aiming at overcoming resistance of tumors in this subset of patients include combining different ICIs with either chemotherapy, VEGF inhibitors, cancer vaccines, adoptive cell transfer, or BTC antibodies. Despite the myriad of strategies being tested, further studies are needed to confirm their efficacy among patients with pMMR-MSI-L tumors that comprise the majority of mCRC disease. We look forward to the results of the ongoing clinical trials presented in this review in hopes that outcomes can be improved for all patients with CRC.

## Figures and Tables

**Table 1:** List of ongoing studies evaluating the use of combination treatments in mismatch repair-deficient microsatellite instability-high colorectal cancers.

Trial type	Trial NCT identifier	Disease burden	Immune checkpoint inhibitor	Study treatment groups
Phase III	NCT02912559	Stage IIICRC	Atezolizumab	Adjuvant atezolizumab + FOLFOX^∗^ versus FOLFOX alone
	NCT02997228	First-line mCRC	Atezolizumab	Atezolizumab versus atezolizumab + FOLFOX + bevacizumab versus FOLFOX + bevacizumab
	NCT02563002	First-line mCRC	Pembrolizumab	Pembrolizumab versus standard-of-care chemotherapy
Phase II	NCT02460198	mCRC: refractory or ≥1 prior therapy	Pembrolizumab	Pembrolizumab
	NCT03150706	mCRC: >1 prior therapy	Avelumab	Avelumab
	NCT02060188	Refractory CRC	Nivolumab ± ipilimumab	Nivolumab ± ipilimumab or daratumumab or anti-LAG3^∗∗^ antibody
Phase I	NCT01633970	Locally advanced or metastatic solid tumors	Atezolizumab	Atezolizumab + Bevacizumab Atezolizumab + Bevacizumab + FOLFOX Atezolizumab + carboplatin + paclitaxel atezolizumab + carboplatin + pemetrexed atezolizumab + carboplatin + nab-paclitaxel atezolizumab + nab-paclitaxel

^∗^FOLFOX: 5-fluorouracil, leucovorin, and oxaliplatin. ^∗∗^LAG3: lymphocyte activation gene 3 protein. Data partially from [235, 249]. Clinical trial details can be accessed at ClinicalTrials.gov database.

**Table 2:** Summary of current strategies being investigated in mismatch repair-deficient microsatellite instability-high colorectal cancers.

Current strategies	Agent (s)	Target (s)	FDA approval Date
Single-agent ICIs	Tremelimumab [143]	CTLA-4	.....
	Nivolumab [144, 146–148]	PD-1	July 2017
	Pembrolizumab [42, 134]		May 2017
	Atezolizumab [10, 43]	PD-L1	.....
	Avelumab [10, 43]		.....
	Durvalumab [10, 43]		.....
ICI + chemotherapy	Bevacizumab + FOLFOX [152]	VEGF for Bevacizumab	.....
	Atezolizumab + FOLFOX [13, 153, 154]	PD-L1 for Atezolizumab	.....
Combinations of ICIs	Durvalumab + Tremelimumab [157, 158]	PD-L1 + CTLA-4, respectively	.....
	Nivolumab + Ipilimumab [147, 159]	PD-1 + CTLA-4, respectively	July 2018

**Table 3:** List of ongoing studies evaluating the use of combination treatments in mismatch repair-proficient microsatellite instability-low colorectal cancer.

Trial NCT identifier	Checkpoint inhibitor	Trial type	Disease burden	Combination treatment	Target (s)
NCT02876224	Atezolizumab	Phase I	mCRC	Cobimetinib + bevacizumab	MEK + VEGFA, respectively
NCT02873195		Phase II	Refractory CRC	Cobimetinib + bevacizumab	MEK + VEGFA, respectively
NCT02788279		Phase III	mCRC	Cobimetinib + regorafenib	MEK + Multi-kinase, respectively
NCT02291289		Phase II	First-line metastatic CRC	Cobimetinib	MEK
NCT02484404	Durvalumab	Phase I/II	Refractory CRC	Cediranib	VEGFR and KIT
NCT02888743	Durvalumab ± tremelimumab	Phase I	mCRC	Radiation	.....
NCT03122509		Phase II	mCRC	Radiation or ablation	.....
NCT03007407		Phase II	mCRC	Radiation	.....
NCT03428126	Durvalumab	Phase II	mCRC	Trametinib	MEK
NCT02811497		Phase II	mCRC	Azacitidine	DNMT
NCT02327078	Nivolumab	Phase I/II	CRC and solid tumors	Epacadostat	IDO1
NCT02948348		Phase I/II	Locally advanced rectal cancer	Chemoradiation	.....
NCT0280546		Phase II	Refractory CRC	TAS-102	.....
NCT02060188	Nivolumab ± ipilimumab	Phase II	Refractory CRC	Cobimetinib + daratumumab	MEK + CD38, respectively
NCT03271047		Phase I/II	Pretreated mCRC	Binimetinib	MEK
NCT03104439		Phase II	CRC arm	Radiation	.....
NCT03377361		Phase I/II	Pretreated mCRC	Trametinib	MEK
NCT03442569		Phase II	RAS-wild-type CRC	Panitumumab	EGFR
NCT03026140		Phase II	Stage I-IIICRC	Celecoxib	COX2
NCT02512172	Pembrolizumab	Phase I	Pretreated mCRC	Oral Azacitidine + romidepsin	DNMT + HDAC1 and/or HDAC2, respectively
NCT03374254		Phase Ib	mCRC	Binimetinib ± FOLFOX or FOLFIRI	MEK for Binimetinib
NCT02856425		Phase I/II	mCRC	Nintedanib	VEGFR, PDGFR, and FGFR
NCT02959437		Phase I/II	Refractory CRC and NSCLC	Azacitidine + epacadostat	DNMT + IDO1, respectively
NCT02713373		Phase Ib/II	Pretreated mCRC	Cetuximab	EGFR
NCT01174121		Phase II	GI tumors and CRC arm	TILs, IL-2, cytoxan, and fludarabine	.....
NCT03374254		Phase II	mCRC	Binimetinib, FOLFOX and FOLFIRI	MEK for Binimetinib
NCT03176264	PDR001	Phase I	First-line metastatic CRC	FOLFOX + bevacizumab	VEGFA for Bevacizumab
NCT03081494		Phase I	Pretreated mCRC	Regorafenib	Multikinase
NCT03258398	Avelumab	Phase II	.....	eFT508	MNK

Data partially from [235, 249]. Clinical trial details can be accessed at ClinicalTrials.gov database.

**Table 4:** Summary of potential combination strategies in mismatch repair-proficient microsatellite instability-low colorectal cancer.

Potential combination strategies		Target (s)
ICIs + RT	RFA or EBRT + pembrolizumab [175]	PD-1 for Pembrolizumab
	RFA + durvalumab + tremelimumab [177]	PD-L1 for Durvalumab
		CTLA-4 for Tremelimumab
ICIs + chemotherapy + anti-angiogenic agents	Atezolizumab + bevacizumab ± FOLFOX [182–184]	PD-L1 for Atezolizumab
		VEGF for Bevacizumab
ICIs + MEK inhibitors	Atezolizumab + cobimetinib [187, 191]	PD-L1 for Atezolizumab
		MEK for Cobimetinib
	Nivolumab + ipilimumab + cobimetinib [193]	PD-1 for Nivolumab
		CTLA-4 for Ipilimumab
		MEK for Cobimetinib
ICIs + MEK inhibitors + anti-angiogenic agents	Atezolizumab + cobimetinib + bevacizumab [192]	PD-L1 for Atezolizumab
		MEK for Cobimetinib
		VEGF for Bevacizumab
ICIs + BTC engaging antibody therapies	Atezolizumab + CEA-BTC antibody [88, 224, 225, 227]	PD-L1 for Atezolizumab
		CEA for CEA-BTC antibody
ICIs + IDO1 inhibitor	Pembrolizumab + indoximod [235]	PD-1 for Pembrolizumab
		IDO1 for Indoximod
	Nivolumab + epacadostat [235]	PD-1 for Nivolumab
		IDO1 for Epacadostat
ICIs + anti-CSF1R antibody	Durvalumab + pexidartinib [238]	PD-L1 for Durvalumab
		CSF1R for Pexidartinib

## Abbreviations

ACT: Adoptive cell transfer
AE: Adverse event
ASCO: American society of clinical oncology
APC: Antigen presenting cell
BSC: Best supportive care
B2M: Beta 2-microglobulin
BTC: Bispecific T-cell
CEA: Carcinoembryonic antigen
CAR: Chimeric antigen receptors
CIN: Chromosomal instability
CRC: Colorectal cancer
CR: Complete response
CTLA 4: Cytotoxic T-lymphocyte associated antigen 4
DC: Dendritic cell
DNA: Deoxyribonucleic acid
DFS: Disease-free survival
EGFR: Epidermal growth factor receptor
EMA: European medicines agency
ESMO: European society for medical oncology
5-FU: 5-Fluorouracil
HR: Hazard ratio
HLA: Human leukocyte antigen
ICI: Immune checkpoint inhibitor
IDO1: Indoleamine 2,3-dioxygenase 1
IFN: Interferon
IL: Interleukin
JAK: Janus Kinase
mCRC: Metastatic colorectal cancer
MSI: Microsatellite instability
MSI-H: Microsatellite instability-high
MSI-L: Microsatellite instability-low
MSS: Microsatellite stable
MMR: Mismatch repair
dMMR: Mismatch repair-deficient
d-MMR-MSI-H: Mismatch repair-deficient microsatellite instability-high
pMMR: Mismatch repair-proficient
pMMR-MSI-L: Mismatch repair-proficient microsatellite instability-low
MEK: Mitogen activated protein kinase
MLH 1: MutL homolog 1
MSH: MutS homolog
Mitogen activated protein kinase
MDSC: Myeloid-derived suppressor cell
NK: Natural killer
NSCLC: Nonsmall cell lung cancer
OR: Objective response
OS: Overall survival
PR: Partial response
PCR: Polymerase chain reaction
PMS 2: Postmeiotic segregation 2
PD-1: Programmed-death 1
PD-L1: Programmed-death ligand 1
PFS: Progression-free survival
QOL: Quality of life
RT: Radiotherapy
rCRC: Refractory colorectal cancer
T-regs: Regulatory T-cells
SD: Stable disease
TAA: Tumor-associated antigen
VEGF: Vascular endothelial growth factor.
